# The complete chloroplast genome of a pantropical legume, *Canavalia rosea*

**DOI:** 10.1080/23802359.2020.1859341

**Published:** 2021-02-03

**Authors:** Ryosuke Imai, Yui Kajita, Takashi Yamamoto, Koji Takayama, Tadashi Kajita

**Affiliations:** a Iriomote Station, Tropical Biosphere Research Center, University of the Ryukyus, Okinawa, Japan; b Hijirigaoka High & Junior High School, Tama University, Tokyo, Japan; c Department of Botany, Graduate School of Science, Kyoto University, Kyoto, Japan

**Keywords:** *Canavalia rosea*, chloroplast, Fabaceae, pantropical plants with sea-drifted seeds

## Abstract

We assembled a complete chloroplast genome of a pantropical legume, *Canavalia rosea* (Fabaceae). The chloroplast genome was 158,059 bp in length that was composed of a 77,752 bp large single copy region, a 18,993 bp small single copy region, and a pair of 30,657 bp inverted repeats. We detected 135 genes that consisted of 90 protein-coding genes, 37 tRNA genes, eight rRNA genes, and three pseudogenes (*rps16* and a pair of *rpl22*).

*Canavalia rosea* (Sw.) DC. (Fabaceae) is one of the representative species of pantropical plants with sea-drifted seeds (Takayama et al. 2006) that have extremely wide ranges of distribution in the tropics and sub-tropics region. Gene flow via sea-dispersal is an intriguing question to understand the formation of such unique distribution of a single plant species, and chloroplast genomes will provide appropriate markers to evaluate gene flow and genetic structure because of their maternal nature of inheritance. Complete chloroplast genomes would also provide useful information to understand the reproductive strategy of the plants. In this study, we constructed the complete chloroplast genome of *C. rosea* using paired-end short-read data.

We collected leaf samples from a cultivated plant in the glasshouse of Iriomote Station, Tropical Biosphere Research Center, University of the Ryukyus. The plant was grown from seeds collected from a wild population of *C. rosea* in Senegal (at Joal-Fadiout on 3rd December 2000) collected by TK (No. 00120304). The voucher specimen is stored in the URO herbarium, University of the Ryukyus. We extracted total DNA from silica-dried leaves using the CTAB method (Doyle and Doyle 1987). Genomic DNA was sequenced with Illumina Hiseq2000. We obtained 22 million 150 bp paired-end reads and removed low-quality nucleotides and reads using the Trimmomatic 0.39.0 (Bolger et al. 2014) with a palindrome clip threshold of 30 and a simple clip threshold of 10. We assembled a chloroplast genome using the GetOrganelles pipeline (Camacho et al. 2009; Bankevich et al. 2012; Langmead and Salzberg 2012; Wick et al. 2015; Jin et al. 2020) and used GeSeq in CHLOROBOX web service (Tillich et al. 2017) for annotation of the chloroplast genome. The chloroplast genome sequence and annotation were submitted to DDBJ (DNA Data Bank of Japan) accession number LC554221.

The total length of the chloroplast genome was 158,059 bp, which is 164 bp shorter than a closely related species, *Canavalia cathartica* Thouars, the chloroplast genome of which is available in GenBank (accession No. NC_047311). The large single copy (LSC) and small single copy (SSC) regions were 77,752 bp and 18,993 bp, respectively. The length of inverted repeats was 30,657 bp. We detected 135 genes including 90 protein-coding genes, 37 tRNA genes, and 8 rRNA genes, and the numbers of genes were the same as *C. cathartica* NC_047311. We also detected three pseudogenes (*rps16* and a pair of *rpl22*). Pseudogenes of *rps16* were found in other individuals of *C. rosea* (unpublished data), which suggests possible allelic gene loss in this species. Pseudogenes of *rpl22* were also reported in other legumes (Gantt et al. 1991). We constructed a phylogenetic tree of Millettioid/Phaseoloid clade based on the method of Zhang et al. (2020) by using 84 coding regions of 15 chloroplast genomes obtained from GenBank. Each gene was aligned by MAFFT 7.4 (Katoh et al. 2002, 2005; Katoh and Toh 2007), then all genes were concatenated by using SeqKit (Shen et al. 2016). A phylogenetic tree obtained by RAxML-NG (Kozlov et al. 2019) with the GTR + G + I model and bootstrap was drawn using Figtree 1.4.2 (Rambaut 2012) 500 times. The phylogenetic tree of *C. rosea* chloroplast genome formed a clade with *C. cathartica*, which is consistent with that of Zhang et al. (2020) and the generic relationships of the phylogenetic tree were consistent with that of Zhang et al. 2020 (Figure 1).

**Figure 1.**
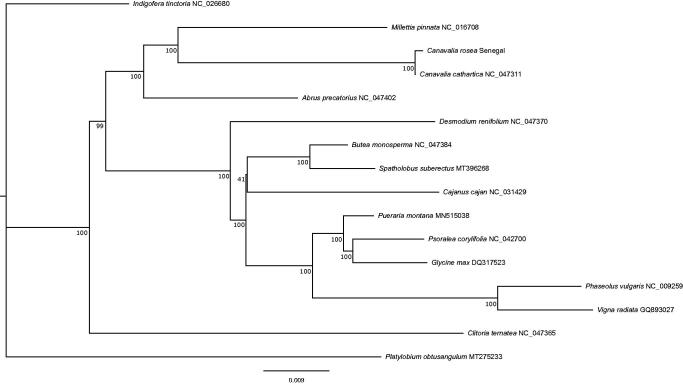
The maximum-likelihood tree of Millettioid/Phaseoloid based on 84 genes in chloroplast genomes. Bootstrap values were shown under branches.

## Data Availability

Data that support the findings of this study are openly available in DDBJ (DNA Data Bank of Japan) and can be accessed at http://getentry.ddbj.nig.ac.jp/top-e.html; accession number LC554221.

## References

[CIT0001] Bankevich A, Nurk S, Antipov D, Gurevich AA, Dvorkin M, Kulikov AS, Lesin VM, Nikolenko SI, Pham S, Prjibelski AD, et al. 2012. SPAdes: a new genome assembly algorithm and its applications to single-cell sequencing. J Comput Biol. 19(5):455–477. 22506599 10.1089/cmb.2012.0021 PMC3342519

[CIT0002] Bolger AM, Lohse M, Usadel B. 2014. Trimmomatic: a flexible trimmer for illumina sequence data. Bioinformatics. 30(15):2114–2120. 24695404 10.1093/bioinformatics/btu170 PMC4103590

[CIT0003] Camacho C, Coulouris G, Avagyan V, Ma N, Papadopoulos J, Bealer K, Madden TL. 2009. BLAST+: architecture and applications. BMC Bioinf. 10(1):421. 10.1186/1471-2105-10-421 PMC2803857 20003500

[CIT0004] Doyle JJ, Doyle JL. 1987. A rapid DNA isolation procedure for small quantities of fresh leaf tissue. Phytochem Bull 19(1): 11–15.

[CIT0005] Gantt JS, Baldauf SL, Calie PJ, Weeden NF, Palmer JD. 1991. Transfer of Rpl22 to the nucleus greatly preceded its loss from the chloroplast and involved the gain of an intron. The EMBO J. 10(10):3073–3078. 1915281 10.1002/j.1460-2075.1991.tb07859.x PMC453023

[CIT0006] Jin J-J, Yu W-B, Yang J-B, Song Y, DePamphilis CW, Yi T-S, Li D-Z. 2020. GetOrganelle: a fast and versatile toolkit for accurate *de novo* assembly of organelle genomes.*Genome Biol*21, 241. 10.1186/s13059-020-02154-5. PMC7488116 32912315

[CIT0007] Katoh K, Toh H. 2007. PartTree: an algorithm to build an approximate tree from a large number of unaligned sequences. Bioinformatics. 23(3):372–374. 17118958 10.1093/bioinformatics/btl592

[CIT0008] Katoh K, Ichi Kuma K, Toh H, Miyata T. 2005. MAFFT version 5: improvement in accuracy of multiple sequence alignment. Nucleic Acids Res. 33(2):511–518. 15661851 10.1093/nar/gki198 PMC548345

[CIT0009] Katoh K, Misawa K, Kuma KI, Miyata T. 2002. MAFFT: a novel method for rapid multiple sequence alignment based on fast Fourier transform. Nucleic Acids Res. 30(14):3059–3066. 12136088 10.1093/nar/gkf436 PMC135756

[CIT0010] Kozlov AM, Darriba D, Flouri T, Morel B, Stamatakis A, Wren J. 2019. RAxML-NG: a fast, scalable and user-friendly tool for maximum likelihood phylogenetic inference. Bioinformatics. 35(21):4453–4455. 31070718 10.1093/bioinformatics/btz305 PMC6821337

[CIT0011] Langmead B, Salzberg SL. 2012. Fast gapped-read alignment with bowtie 2. Nat Methods. 9(4):357–359. 22388286 10.1038/nmeth.1923 PMC3322381

[CIT0012] Rambaut A. 2012. FigTree v1. 4. 2, A Graphical Viewer of Phylogenetic Trees. Available from 〈http://tree.bio.ed.ac.uk/software/figtree/〉

[CIT0013] Shen W, Le S, Li Y, Hu F. 2016. SeqKit: a cross-platform and ultrafast toolkit for FASTA/Q file manipulation. PLoS One. 11(10):e0163962. 27706213 10.1371/journal.pone.0163962 PMC5051824

[CIT0014] Takayama K, Kajita T, Murata J, Tateishi Y. 2006. Phylogeography and genetic structure of *Hibiscus tiliaceus* - speciation of a pantropical plant with sea-drifted seeds. Mol Ecol. 15(10):2871–2881. 16911207 10.1111/j.1365-294X.2006.02963.x

[CIT0015] Tillich M, Lehwark P, Pellizzer T, Ulbricht-Jones ES, Fischer A, Bock R, Greiner S. 2017. GeSeq - versatile and accurate annotation of organelle genomes. Nucleic Acids Res. 45(W1):W6–W11. 28486635 10.1093/nar/gkx391 PMC5570176

[CIT0016] Wick RR, Schultz MB, Zobel J, Jolt KE. 2015. Bandage: interactive visualization of *de novo* genome assemblies. Bioinformatics. 31(20):3350–3352. 26099265 10.1093/bioinformatics/btv383 PMC4595904

[CIT0017] Zhang R, Wang Y-H, Jin J-J, Stull GW, Bruneau A, Cardoso D, De Queiroz LP, Moore MJ, Zhang S-D, Chen S-Y, et al. 2020. Exploration of plastid phylogenomic conflict yields new insights into the deep relationships of leguminosae. Syst Biol. 69(4):613–622. 32065640 10.1093/sysbio/syaa013 PMC7302050

